# There Is No Joy like Malicious Joy: Schadenfreude in Young Children

**DOI:** 10.1371/journal.pone.0100233

**Published:** 2014-07-02

**Authors:** Simone G. Shamay-Tsoory, Dorin Ahronberg-Kirschenbaum, Nirit Bauminger-Zviely

**Affiliations:** 1 Department of Psychology, University of Haifa, Haifa, Israel; 2 School of Education, Bar Ilan University of Haifa, Haifa, Israel; University of Tuebingen Medical School, Germany

## Abstract

Human emotions are strongly shaped by the tendency to compare the relative state of oneself to others. Although social comparison based emotions such as jealousy and schadenfreude (pleasure in the other misfortune) are important social emotions, little is known about their developmental origins. To examine if schadenfreude develops as a response to inequity aversion, we assessed the reactions of children to the termination of unequal and equal triadic situations. We demonstrate that children as early as 24 months show signs of schadenfreude following the termination of an unequal situation. Although both conditions involved the same amount of gains, the children displayed greater positive expressions following the disruption of the unequal as compared to the equal condition, indicating that inequity aversion can be observed earlier than reported before. These results support an early evolutionary origin of inequity aversion and indicate that schadenfreude has evolved as a response to unfairness.

## Introduction

The developmental origins and proximate mechanisms behind social comparison based emotions are not well understood, despite recent progress (e.g. [1]). Social comparison based emotions involve two (or more) person situations in which one's emotions depends on the other's state [2]. The process of social comparison may trigger prosocial emotions such as empathy and compassion to the distress of others but also competitive emotions such as malicious joy or schadenfreude when facing others misfortune [3]. Schadenfreude is a relatively unstudied emotion and involves experiencing pleasure when another person faces an unfavorable event [4]. Schadenfreude is related to other competitive social comparison based emotions such as envy [5] and resentment [6] and it frequently arises in situations in which the target deserves the misfortune (e.g. [7], [8]). Interestingly, while there is strong evidence for biological, evolutionary and developmental roots of prosocial empathically motivated helping behaviors (e.g. [9]) the evolutionary and developmental origins of schadenfreude are unknown.

One possibility is that schadenfreude, as well as other competitive social comparison based-emotions such as envy and jealousy, originally evolved, as a response to competition between rivals over limited resources. According to this notion, schadenfreude involves pleasure associated with gains in the context of limited resources. For example, siblings—who from conception are rivals for a parent's resources [10] may experience schadenfreude, as a response to a potential reward such as parental availability. Thus, the suffering of the sibling may be rewarding because it signals potential additional parental resources. Sibling rivalry is frequently reported in the animal kingdom, including sibling murder between baby eaglets and pelicans [11] or between shark embryos [12], indicating that it has an evolutionary importance.

Similarly to sibling rivalry, mating rivalry may have evolved as a response to competition between same-sex individuals—who are rivals for mating partners. It has been shown that mating strategies in both men and women includes derogating other individuals as a basic mechanism for increasing self-attractiveness [13]. Based on these findings it has been proposed that schadenfreude is a psychological mechanism that responds to misfortunes that lower competitors' mate value in order to increase mating opportunities [14].

Thus, the sibling and mating rivalry accounts of schadenfreude may indicate that the distress of a rival (e.g. same-sex rival; sibling) is rewarding as it indicates a potential increase in resources such as parental attention or mating partners. The sibling and the mating rivalry accounts of schadenfreude are in line with the ‘gain’ hypothesis, according to which, schadenfreude is viewed as an emotion that originates from competition over limited recourses and therefore it involves a positive reaction to a potential gain during competition [15]. According to this theoretical formulation, pleasure, is a basic automatic reaction to positive rewards and malicious pleasure is the result of the potential reward rather than pleasure in the other's misfortune [15]. This suggests that schadenfreude involves a positive reaction to potential gains which may be unrelated to the suffering of the rival. Thus, if indeed, as suggested by the gain hypothesis, schadenfreude is a response to a potential gain regardless of a rivals' misfortune, than it should involve similar amounts of positive reactions in response to the termination of a competitive situation vs. a non-competitive situation if both situations involve similar amounts of gains.

Yet, an equal plausible hypothesis suggested here is that schadenfreude has evolved as a response to inequity aversion or the resistance to unfairness and inequalities. Inequity aversion predicts that individuals are sensitive to how their payoffs compare with those of others and therefore individuals may react negatively to unfair treatment [16]. According to this, schadenfreude may involve the pleasure of termination of an unpleasant unequal situation. Interestingly, it has been shown that inequity aversion develops early in children, further attesting to its evolutionary significance. Fehr, Bernhard, and Rockenbach [17] have reported that children at age 7–8 prefer resource allocations that remove advantageous or disadvantageous inequality. Other studies suggest that inequity aversion may be observed even before the age of five. It has been shown that children as young as four years old can judge situations to be undesirable based on concerns with fairness (for reviews, see [18], [19], [20]). In addition, Paulus, Gillis, Li, & Moore, [21] reported that preschool children involve third parties in dyadic sharing situations. Moreover, LoBue, Nishida et al., [22] have recently reported that even three years old children react negatively to disadvantageous inequality. Other reports show that even 15-month-old infants are sensitive to fairness and can engage in altruistic sharing [23].

That inequity aversion is evident early indicates that it has deep developmental roots. It has been suggested that negative reactions to an unequal reward distribution in regard to the effort invested may have been essential for the evolution of cooperation [24]. Indeed, negative reactions to inequalities have been reported not only in human adults but also in capuchin monkeys [25] and domestic dogs [26].

Considering the evolutionary significance of negative reactions to disadvantageous distribution, it is possible that schadenfreude has evolved as a positive reaction to the termination of inequity.

To test this hypothesis, in the current study we examined the emotional reactions to equal and unequal conditions in the distribution of parental attention in two and three years of children. Schadenfreude has been rarely reported in children and the only study that directly measured it reported signs of schadenfreude in 7 years old children which decreased with age [1].

It was reasoned that if schadenfreude is an emotion that originates from inequity aversion then the termination of an unequal condition should trigger more positive reactions as compared to the termination of an equal event even if the two conditions involve equal gains. Thus, we placed two years old children in a real social situation involving their mother and a peer. As opposed to previous studies which manipulated envy to provoke schadenfreude, in the current study we manipulated jealousy to elicit schadenfreude. Jealousy is the emotion children experience in a triadic situation, when there is a potentially unequal situation which raises a concern about losing exclusivity in significant relationships to a third party (e.g. [27]). In contrast, envy may involve only two-person situations, and this feeling comprises the wish to have another person's possession or success and/or the wish that the other person did not possess this desired characteristic or object [28]. Whereas envy and jealousy are somewhat different [5], these emotions are related and often co-occur [29], [30] indicating that jealousy could equally be associated with schadenfreude.

The study included two main conditions each comprised of two phases. In each condition, in the first phase the mother read a book, while in the second phase the mother accidently spilled water over the book. In the unequal condition (UNEQUAL) the mother read the book to the similar-aged peer (jealousy manipulation phase) while in the equal condition (EQUAL) the mother read the book to herself. We sought to examine if two- and three-year old children can show signs of schadenfreude following the termination of the jealousy phase (UNEQUAL) as compared to the control (EQUAL) condition.

## Methods

The research has been approved by the University of Haifa Ethic committee. We contacted the parents of the children through ads, following the approval of the University of Haifa ethics committee. After obtaining written parental consent for participation, we advised the parents about the nature of the research by telephone. To reduce stress and use an ecologically valid environment, all experimental conditions were carried out in the home of the target child.

### Participants

105 participants participated in the study. The participants included 35 triads including a mother (mean age = 35.486, SD = 4.461) her child (20 girls, 15 boys; mean age = 3.050, SD = 0.650) and a similar-aged peer (22 girls, 13 boys; mean age = 3.485,SD = 4.461).

### Task

#### The EQUAL condition: Phase 1: story-reading scenario

**Figure 1:**
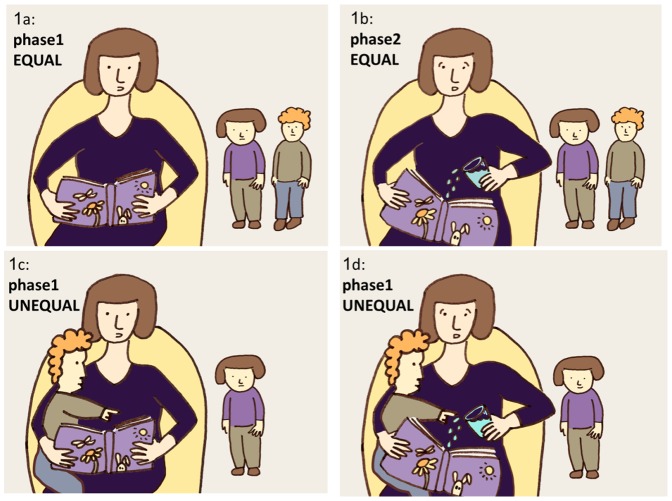
The EQUAL and the UNEQUAL conditions. In the EQUAL condition the mother reads a book aloud to herself while the kids are playing (Figure 1a) the mother is then signaled to take the glass of water and accidently spill water over the book (Figure 1b). In the UNEQUAL condition the mother placed the peer on her lap and embraced the child while reading a story aloud to that child (Figure 1c) and then she was signaled to accidently spill water on the book (Figure 1d). At both conditions the child were allowed to play freely.

Based on Bauminger, Chomsky-Smolkin et al. [31], the story-reading scenario included a triad comprising the target child, the mother and a peer who was a familiar preschool classmate. The session began with the mother sitting on a chair near a table on which a book and a glass of water were placed. The experimenter encouraged the two children to play with the age-appropriate toys and instructed the mother to ignore the children while completing a demographic questionnaire (2 min). As depicted in Figure 1a, upon the experimenter's signal, the mother took the book from the table and started reading the story aloud to herself (2 min).

#### The EQUAL condition: Phase 2: spilled water scenario

At the end of the 2 min, or if the target child showed substantial distress before that time, the mother was signaled to take the glass of water and accidently spill water over the book (Figure 1b).

#### The UNEQUAL condition: Phase 1: story-reading scenario

The session began with the mother sitting on a chair near a table on which a book and a glass of water were placed. Upon the experimenter's signal, the mother placed the peer on her lap and embraced the child while reading a story aloud to that child (Figure 1c).

#### The UNEQUAL condition: Phase 2: spilled water

At the end of the 2 min, or if the target child showed substantial distress before that time, the mother was signaled to take the glass of water and accidently spill water on the book(Figure 1d).

At the end of the experiment, the mother invited the target child to sit on her lap and hear the story.

The water spill manipulation was used to provoke schadenfreude as this emotion is frequently provoked following a misfortunate termination of a competitive situation [5]. Therefore, it was predicted that spilling water over the book following the unequal situation (reading the book to a peer) would provoke schadenfreude.

In the EQUAL situation (control condition) the mother took the book from the table and started reading the story aloud to herself, while in the UNEQUAL situation (experimental condition) the mother placed the peer on her lap and embraced the child while reading a story aloud to him/her. It should be noted that the control condition was designed to be as similar as possible to the experimental condition. It was reasoned that if the mother would not read the story aloud to herself than the children would not notice that she is reading a story. In both the experimental and control conditions both children could listen to the story. The only difference between the conditions was that in the experimental condition the peer was sitting on the mother's lap while she was reading the story.

#### Coding systems for jealousy and schadenfreude

Children's videotaped jealousy and schadenfreude-provoked behaviors, verbalizations, and affects were assessed using three coding scales: hierarchical explicitness of emotional reaction, quantity of jealousy and schadenfreurde behaviors and affect. These measures provide a comprehensive assessment of children's real-time emotional reaction and the amount of these reactions. The jealousy scales were the same scales used in previous studies [31], [32], [33] derived from the behaviors, verbalizations, and affects identified as jealousy indices by previous research (e.g. [34], [35]). The schadenfreude ratings were novel and developed for the current study based on the validated jealousy ratings. Basically, the schadenfreude ratings were parallel and equivalent to the jealousy ratings.

#### Explicitness of the emotional response

The explicitness of the emotional responses in Phase 1 (story reading) and phase 2 (spilled water) of the EQUAL and UNEQUAL conditions were coded according to the following scales:

- *Phase 1* *Hierarchical jealousy scale*: This 7-point scale ranked explicitness of actions, verbalizations, and affective expressions of jealousy and in hierarchical order, from no interest at all (1) up to direct indication of the children's comparison and lack of equality, accompanied by negative affect (7) [see 31 for detailed descriptions of this scale], [32]. Coders assigned the child the highest score evidenced over the 2-minute scenario. A score of 4 and above indicated explicit actions (e.g., pushing the rival aside and standing between the mother and peer), verbalizations (e.g., “I want too”), and affects (e.g., shouting “Enough!”) that reflected jealousy, whereas a score below 4 indicated only eye gaze in different degrees.
- *Phase 2* *Hierarchical schadenfreude scale:* This 7-point scale was based on the jealousy phase and ranked explicitness of actions, verbalizations, and affective expressions of schadenfreude in hierarchical order, from no interest at all (1) up to direct indication of the children's comparison and lack of equality, accompanied by positive affect (7). It is important to note that differently from the hierarchical jealousy scale here, the emotions that were coded were positive affect rather than negative emotions that were coded in the jealousy condition. Coders assigned the child the highest score evidenced over the 2-minute scenario. A score of 4 and above indicated explicit actions (e.g., jumping with happiness), verbalizations (e.g., “yes! The water spilled over the book!”), and affects (e.g., shouting “great!”) that reflected schadenfreude, whereas a score below 4 indicated only eye gaze in different degrees.

#### Quantity of different jealousy and schadenfreude manifestations

The quantity of the emotional responses in Phase 1 (story reading) and phase 2 (spilled water) of the EQUAL and UNEQUAL conditions were coded according to the following scales:

- *Phase 1: Behavioral jealousy coding category scale:* This scale assessed the frequency of jealousy manifestations comprising two main categories: (1) verbalizations, including attention-seeking comments (e.g., “I don't feel good”) and interactive comments (e.g., repeating words from the story being read or answering questions aimed at the peer); and (2) actions including attention seeking actions (e.g., caressing mom's hair) and involvement actions (e.g., putting one's head between the book and the peer, to block the peer's view). Scores were calculated for each category and were divided by scenario duration, with higher scores indicating a higher quantity of jealousy manifestations.
- *Phase 2: Behavioral schadenfreude coding category scale*: As in the jealousy scale, this scale assessed the frequency of schadenfreude manifestations comprising two main categories: (1) verbalizations (e.g.,“good”) and interactive comments (e.g., “can you read it to me now?”); and (2) actions including jumping, clapping hands, running, rolling on the floor. Scores were calculated for each category and were divided by scenario duration, with higher scores indicating a higher quantity of schadenfreude manifestations.

#### Affect scale

The affective changes in Phase 1 (story reading) and phase 2 (spilled water) of the UNEQUAL conditions were coded according to the following scales:

- *Phase 1:* Based on Bauminger-Zvieli and Kugelmass [33], this 4-point scale was designed to assess a possible change in children's negative affect before versus during the jealousy provoking social scenario. Coding, ranging from 1 (very negative affect) to 4 (very positive affect), was executed twice: Time 1 - when the peer-rival entered the room and each child played alone with his/her toys; Time 2 – when the mother took the peer onto her lap and read him/her a story.
- *Phase 2:* To assess a possible change in children's positive affect before versus during the schadenfreude provoking social scenario we used a similar to phase 1 affect scale. Coding, ranging from 1 (very positive affect) to 4 (very negative affect), was executed twice: Time 1 - when the mother read the story to the peer/herself (few seconds after Time 2 in phase 1); Time 2 – when the mother spilled the water over the book.

It should be noted that the scoring system was based on Bauminger-Zvieli and Kugelmass [33]. This scoring method does not allow measuring change in affect during the EQUAL situation as Time 2 is missing in this scenario (the mother does not take a peer onto her lap).

All videotapes underwent coding by two coders who separately in a counterbalancing order assigned scores to each child. The interclass correlation coefficients for the mother scenario were 0.90 for all jealousy and schadenfreude categories (verbalization, action). In the few cases of disagreement between the coders, the value used for data processing was the mean of the two coders' scores for that child.

#### Assessment of Children's Spontaneous Schadenfreude Expressions Reported by Mothers

To examine the relationship between the behaviors coded during the experiment and the ratings of the mothers of schadenfreude within the natural home environment, we developed for the current study a scale which asked “Has your child ever expressed jealousy/schadenfreude?” (yes/no)

## Results

### Explicitness: Hierarchical jealousy and schadenfreude scales

**Figure 2:**
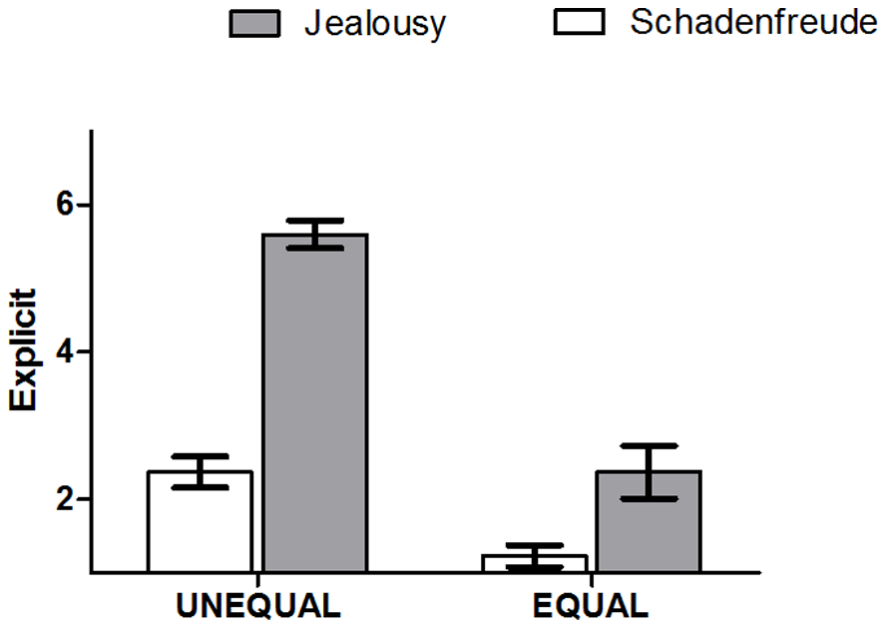
Explicitness: Hierarchical jealousy and schadenfreude scales. A significant main effects of the factors *phase,condition* and phase*condition. *F*ollow-up paired t tests indicate that the difference between the UNEQUAL and the EQUAL condition was evident both in phase 1 ratings and in phase 2 ratings.

In order to assess the difference in the change in the explicitness of emotional ratings between the UNEQUAL and EQUAL conditions in phase 1 and 2, we performed an ANOVA of the rating data, testing for a significant interaction of phase (1,2)*condition (EQUAL, UNEQUAL). This ANOVA showed significant main effects of the factors *phase* (*F(1,34) = 81.749, P<0.0001*) and *condition* (*F(1,34) = 114.750, P<0.0001*). Significant interaction was found for phase*condition (*F(1,34) = 26.046, P<0.0001*). The main effect *phase* resulted from higher ratings for phase 1 (phase 1: *M/S.E. = 3.986/0.189*; phase 2 = *1.323/0.214*), the main effect of *condition* resulted from higher ratings in the UNEQUAL condition (UNEQUAL = *3.986/0.204*; EQUAL = *1.323/0.151*). As shown in Figure 2, follow-up paired t tests indicated that the difference between the UNEQUAL and the EQUAL condition was evident both in phase 1 ratings (*t (34) = 14.358, P = 0.0001*) and in phase 2 ratings (*t (34) = 3.353, P = 0.002*).

### Quantity of different emotional manifestations

**Figure 3:**
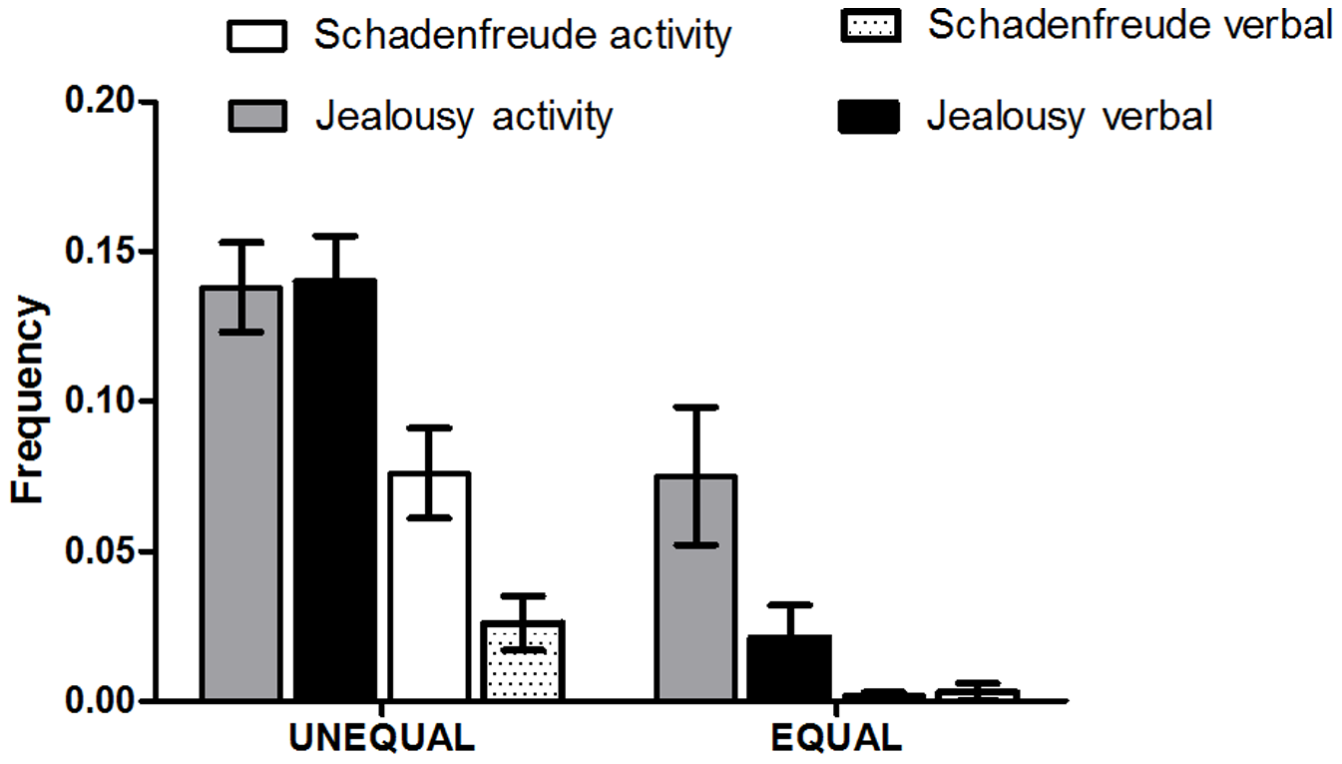
Quantity of different emotional manifestations. A significant interaction of phase (phase 1, phase 2)*condition (EQUAL, UNEQUAL) * category (verbalization/action). Follow-up paired t tests indicated that the differences between the UNEQUAL and the EQUAL condition were evident in the action phase 1 ratings, verbalization phase 1ratings, in the phase 2 action ratings and phase 2 verbalization ratings.

An ANOVA of the quantity data was carried out testing for a significant interaction of phase (phase 1, phase 2)*condition (EQUAL, UNEQUAL) * category (verbalization/action). This ANOVA showed significant main effects of the factors *phase* (*F(1,34) = 24.706, P<0.0001*), *category* (*F(1,34) = 11.617, P<0.002*) and *condition* (*F(1,34) = 44.182, P<0.0001*). Significant interaction effects were found for phase*condition (*F(1,34) = 7.481, P<0.01*) and phase*condition* category (*F(1,34) = 19.001, P<0.0001*). As shown in Figure 3, the main effect *phase* resulted from higher frequency ratings for phase 1, the main effect of *condition* resulted from higher ratings in the UNEQUAL condition and the main effect for the *category* resulted from higher ratings of actions. Follow-up paired t tests indicated that the differences between the UNEQUAL and the EQUAL condition were evident in the action phase 1 ratings (*t (34) = 2.811, P<0.008*), verbalization phase 1ratings (*t (34) = 7.417, P<0.0001*), in the phase 2 action ratings (*t (34) = 4.964, P<0.0001*) and phase 2 verbalization ratings (*t (34) = 2.829, P<0.008*).

### Affect scale

**Figure 4:**
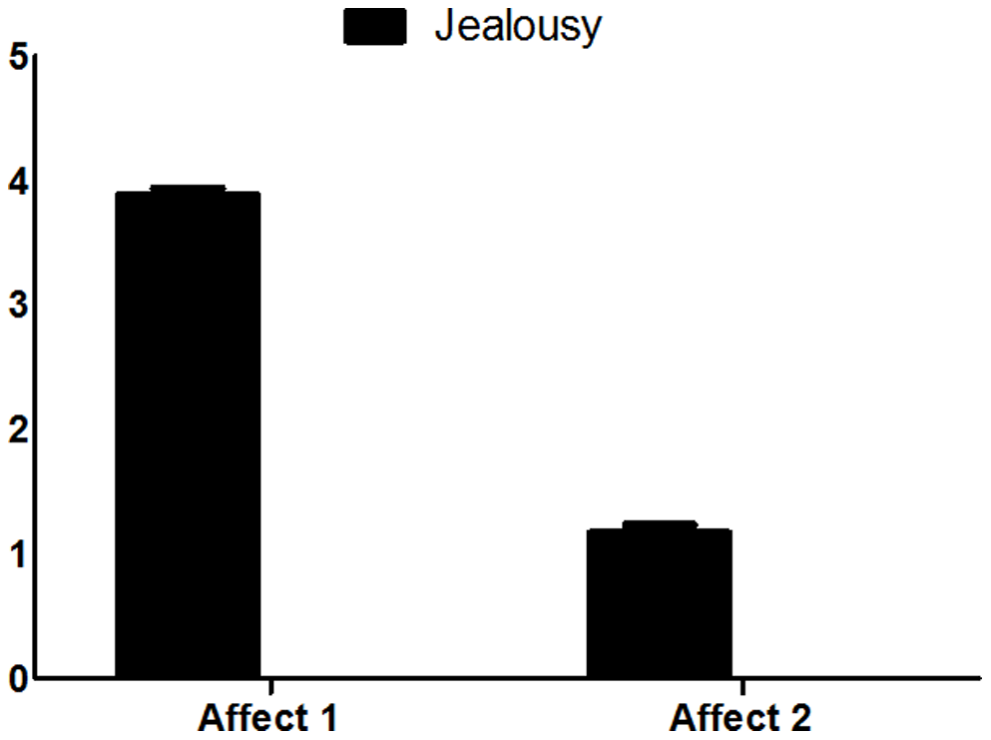
Affect scale. A significant change in negative affect (reduced positive affect) in the jealousy condition.

**Figure 5:**
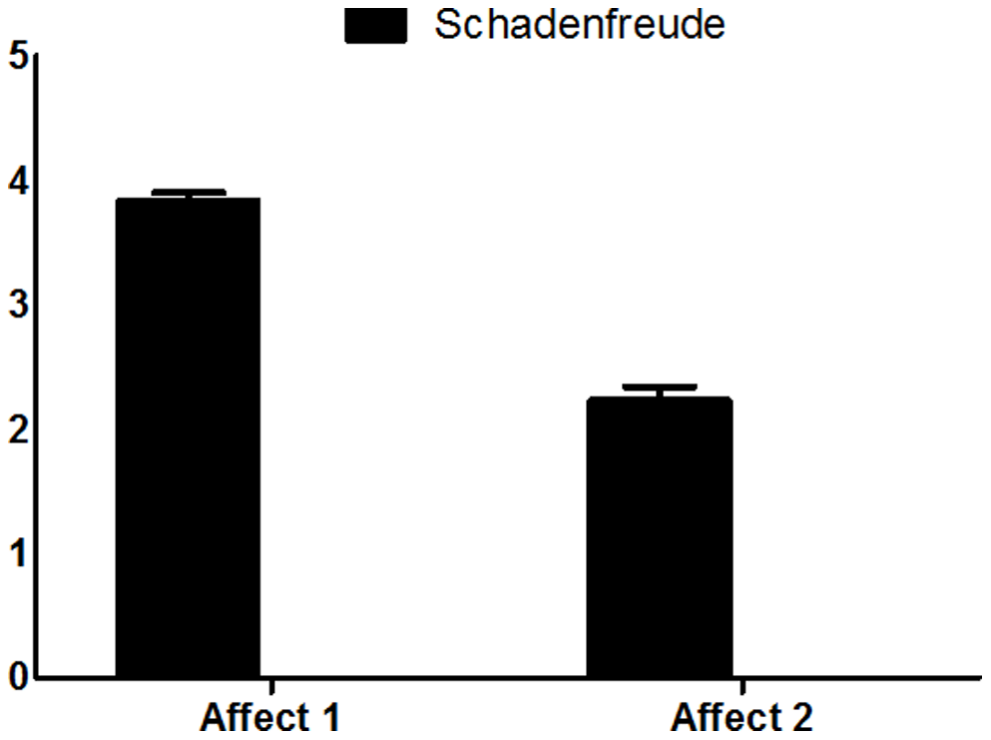
Affect scale. A significant change in negative affect (reduced negative affect) in the schadenfreude condition.

As indicated above this scale assesses the change in affect before versus during the jealousy (phase 1) and schadenfreude (phase 2) provoking social scenario. In order to assess the difference in the negative emotional manifestations between the *affect 1* and *affect 2* in the phase 1, we performed a paired t-test which indicated a significant change in negative affect (*t (34) = 16.139, P<0.0001*) (Fig. 4). Similarly a significant change in positive affect was found in phase 2(*t (34) = 11.662, P<0.0001*), indicating increase positive affect following the spilled water condition (Fig. 5).

### Parents ratings

We carried out a MANOVA to compare the emotional responses measured in phase 2 (explicitness of emotional response, frequency of action and verbal, affect 2 phase 2) following the UNEQUAL condition and compared the reactions of children reported by their mother to have expressed schadenfreude (N = 25) to those reported not to have expressed schadenfreude in the natural home environment (N = 10). This MANOVA showed a significant effect (*F(4,30) = 5.056, P<0.002*), indicating an overall significant difference between the groups. Tests of between subjects effects indicated significant differences between the groups in the variables affect 2 phase 2 (*F(1,33) = 15.714, P<0.0001*) and activity (*F(1,33) = 15.714, P<0.018*) but not for the variables frequency of verbal responses (*F(1,33) = 2.11, P = 0.156*) and explicitness of the response (*F(1,33) = 1.911, P = 0.176*).

Finally, we carried out a MANOVA to compare the emotional responses measured in phase 2 (explicitness of emotional response, frequency of action and verbal, affect 2) following the UNEQUAL condition in same sex peers (N = 21) and different sex peers (N = 14). This MANOVA showed a non-significant effect (*F(4,30) = 0.948, P = 0.450*), indicating no overall significant difference between the groups.

## Discussion

The current study examined if jealousy towards a peer would influence schadenfreude when the peer experiences a subsequent misfortune event. In contrast, the same event occurring without jealousy was not expected to produce schadenfreude.

According to the ‘gain’ hypothesis, schadenfreude is viewed as a positive reaction to a potential reward. This hypothesis suggests that while in most circumstances observing others in physical or emotional pain lead to empathy (e.g. [9], [36], [37]), in competitive zero-sum situations we may gain from misfortunes befalling on another individual which may lead to schadenfreude [15].

In the current study, the EQUAL as well as the UNEQUAL conditions ended similarly, the water spill phase in both conditions resulted in potentially more maternal attention. Yet, children reacted with greater emotional intensity following the UNEQUAL as compared to the EQUAL condition. Furthermore, children did not show more schadenfreude toward same sex targets as compared to opposite sex target further contradicting the model of Colyn and Gordon [38], which suggests that schadenfreude is a psychological mechanism that responds to misfortunes that lower competitors' mate value in order to increase mating opportunities.

The analyses of the hierarchical scales and quantity of different emotional manifestations scales show differences between the UNEQUAL and EQUAL conditions indicating inequality was associated with higher emotional ratings as compared to equality. While inequity produced higher jealousy ratings than equity, the termination of the inequitable situation produced higher schadenfreude ratings as compared to the termination of the equitable situation. Furthermore, the analysis of the affect scale shows a significant decrease in positive affect in the jealousy manipulation and a significant decrease in negative affect following the schadenfreude manipulation. The difference between schadenfreude ratings in the UNEQUAL and EQUAL conditions throughout the scales indicates that indeed the termination of the inequitable situation provokes schadenfreude. Furthermore, that signs of schadenfreude were observed even in the youngest children in the sample around the age of 24 months, both support the hypotheses of early evolutionary origin of inequity aversion and indicate that schadenfreude may have evolved as a response to unfair allocation of resources. Furthermore, the findings showing that children reported by their parents to have shown signs of schadefreude at home obtained higher schadenfreude ratings in the UNEQUAL condition as compared to children not reported to have shown signs of schadenfreude, further confirm that indeed the positive emotional reactions observed following the UNEQUAL condition reflect emotional reactions associated with schadenfreude.

It should be noted that while some reports on inequity aversion have found sensitivity to inequity around the age of 7 [17], others have reported that children as young as 3 years old react negatively to advantageous or disadvantageous inequality [22]. One possibility of the differences in the findings is the different methods used. While Fehr et al. [17] examined how children allocate rewards between themselves and another random partner, Lebou et al., [22] have probed the emotional reactions of children to the distribution of unequal of rewards made by others. Interestingly, although these studies used similar methods to paradigms used in the research on schadenfreude these emotions are not addressed. Thus, the current findings extend the literature on fairness and inequity aversion by putting forward the role of emotional reactions that emerges following unfair conditions.

Another interesting finding that emerged from the analysis was that jealousy ratings were higher than schadenfreude ratings, suggesting that jealousy is highly intense as compared to schadenfreude. Indeed, it has been suggested that jealousy is more intense than other social comparison based emotions such as envy [5], [39] perhaps because it involves an extreme fear of loss of maternal attention. Research on jealousy shows that this emotions appears most intensely in the majority of children between approximately 13 to 25 months [34] and can be clearly observed around the third year of life [31]. Moreover, there are even reports of forms of jealousy in babies as young as 6 months old [40], further indicating that jealousy is a powerful emotion that develops extremely early in life. Another possibility is that greater responses to negative events are related to a more basic negativity bias which refers to the psychological phenomenon by which humans pay more attention to and give more weight to negative rather than positive information [41]. Hence, the adaptive nature of negativity bias is such that jealousy in response to unfavorable comparison is likely to motivate specific behaviors for eliminating the gap between the self and the other, whereas there is little in the way of response warranted by the favorable comparison.

Finally, although the current study appears to support the hypothesis according to which schadenfreude is related to inequity aversion and not to actual potential gains, it is possible that the two hypotheses are not mutually exclusive.

Although, the termination of both the EQUAL and the UNEQUAL conditions resulted in similar potential reward, the termination of the UNEQUAL condition involved also the elimination of jealousy and therefore there was an additional emotional gain involved. Furthermore, it has been suggested that there are potential social comparison benefits behind any misfortune to the extent that it represents downward comparison and the boost to self-evaluation that might follow [5]. Jealousy, like envy represents the polar opposite of a downward comparison and therefore a misfortune befalling on someone we are jealous of, reverses the unfavorable comparison and may have an ameliorating effect on self-esteem [42].

Collectively, the current study shows for the first time that children as early as 24 months show signs of schadenfreude following the termination of an unequal situation, indicating that inequity aversion can be observed earlier than reported before. These findings imply that social comparison and sensitivity to fairness develop early in life further highlighting the evolutionary significance of positive reactions to the termination of an unfair situation. Furthermore, it has been reported that social comparison based emotions are related to different personality traits including self-esteem, neuroticism and sense of inferiority. Smith et al. [5], for example, reported that dispositional envy is negatively correlated with measures of self-esteem and positively related to depression [5]. Considering the strong relationship between envy, jealousy and schadenfreude, it is possible that individuals with low self-esteem may experience more schadenfreude. Future research may use the paradigm reported here and examine if individual differences in the tendency to feel schadenfreude among young children predicts different personality traits including low self-esteem and neuroticism.
