# Correction: There Is No Joy like Malicious Joy: Schadenfreude in Young Children

**DOI:** 10.1371/journal.pone.0111415

**Published:** 2014-10-13

**Authors:** The PLOS ONE Staff

Figure 1 is incorrect. Figure 1d should read “phase2.” The authors have provided a corrected version here.

**Figure 1:**
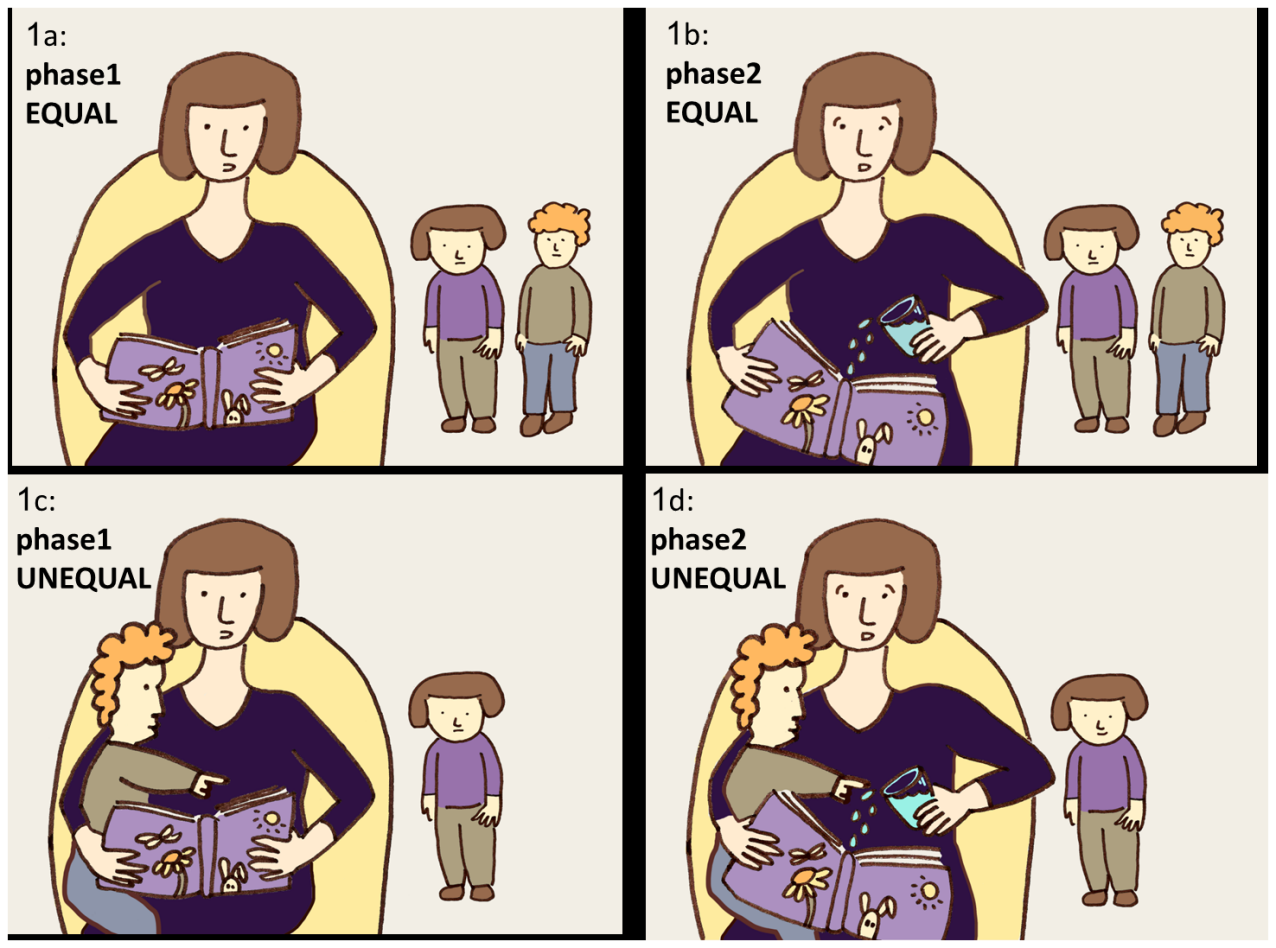
The EQUAL and the UNEQUAL conditions. In the EQUAL condition the mother reads a book aloud to herself while the kids are playing (Figure 1a) the mother is then signaled to take the glass of water and accidently spill water over the book (Figure 1b). In the UNEQUAL condition the mother placed the peer on her lap and embraced the child while reading a story aloud to that child (Figure 1c) and then she was signaled to accidently spill water on the book (Figure 1d). At both conditions the child were allowed to play freely.
